# Assessment of the Airway Characteristics in Children with Cleft Lip and Palate using Cone Beam Computed Tomography

**DOI:** 10.5005/jp-journals-10005-1324

**Published:** 2016-04-22

**Authors:** Anirudh Agarwal, Nikhil Marwah

**Affiliations:** 1 Professor and Head, Department of Orthodontics, Jaipur Dental College, Jaipur Rajasthan, India; 2 Professor and Head, Department of Pedodontics, Mahatma Gandhi Dental College Jaipur, Rajasthan, India

**Keywords:** CBCT, Cleft lip, Cleft palate, Nasopharyngeal.

## Abstract

**Objective:** The aim of our study is to use cone beam computed tomography (CBCT) to assess the dimensional changes in the nasopharyngeal soft-tissue characteristics in children of Indian origin with repaired cleft lip and palate (CLP) and to compare the results with patients with ideal occlusion.

**Materials and methods:** A sample of 20 children (10 girls, 10 boys) with repaired CLP was selected. Cone beam computed tomography scans were taken to measure the nasopharyngeal airway changes in terms of linear measurements and sagittal cross-sectional areas. Error analysis was performed to prevent systematic or random errors. Independent means t-tests and Pearson correlation analysis were used to evaluate sex differences and the correlations among the variables.

**Results:** Nasopharyngeal soft-tissue characteristics were different in the control and the study groups. Subjects with repaired CLP had lesser lower aerial width, lower adenoidal width and lower airway width. The upper airway width was also significantly lesser. The retropalatal and the total airway area were significantly greater in the control group.

**Conclusion:** The narrow pharyngeal airway in patients with CLP might result in functional impairment of breathing in patients. Further investigations are necessary to clarify the relationship between pharyngeal structure and airway function in patients with CLP.

**How to cite this article:** Agarwal A, Marwah N. Assessment of the Airway Characteristics in Children with Cleft Lip and Palate using Cone Beam Computed Tomography. Int J Clin Pediatr Dent 2016;9(1):5-9.

## INTRODUCTION

Recent studies have demonstrated significant differences in facial structures and growth associated with cleft lip and palate (CLP)^1^ compared with those in normal subjects; patients with CLP have a smaller upper airway compared with normal controls.^2,3^ Parents of children with CLP have often reported that their children snore and breathe noisily during sleep, and patients with reduced nasal airways are also predisposed to mouth breathing.^4-6^ Rose et al^7^ found that patients with cleft palate had significantly elevated incidences of mouth breathing, snoring and hypopnea during sleep. These clinical findings are considered to represent the initial symptoms of sleep-disordered breathing.

The high risk for sleep-disordered breathing in children with CLP is caused by the dysfunction of muscles controlling the soft palate in conjunction with structural abnormalities of the maxilla and the mandible.^2^ Patients suffering from sleep-disordered breathing are at increased risk for hypertension, cardiovascular and cerebrovascular diseases and excessive daytime sleepiness.^8^

Morphometric evaluation of the pharyngeal airway is, therefore, important in patients with CLP. Most previous evaluations have been performed by identifying landmarks on lateral cephalometric images and measuring standard lengths and areas in the pharyngeal region.^3,9,10^

Lateral cephalograms are limited by the inherent errors accompanying the two-dimensional (2D) representation of a three-dimensional (3D) structure, including distortion, differences in magnification and the superposition of the bilateral craniofacial structures.^11^

With the advent of low-radiation, rapid computed tomography (CT) scanning,^12-16^ the potential for orthodontists to assess craniofacial growth in 3D is now available,^17,18^ and with that analysis is the capability of evaluating the complete airway.^19,20^

The aim of this study is to assess nasopharyngeal, aerial and adenoidal soft-tissue characteristics in patients with CLP and to compare the results with patients with ideal occlusion.

## MATERIALS AND METHODS

A sample of 40 children was selected with no previous orthodontic treatment, 10 girls and 10 boys each in class I (control) and cleft (study) group. In the study, group 14 patients had unilateral cleft and 6 had bilateral cleft of the palate.

Exclusion criteria were (1) a history of treatment for sleep-disordered breathing, including tonsillectomy, adenoidectomy or recurrent tonsillitis; (2) frequent colds (six or more per year) or (3) a history of dysphagia and continuous positive airway pressure therapy. A further exclusion criterion for the control groups was any type of syndrome. All control subjects had normal craniofacial morphology with no jaw deformities.

**Fig. 1:**
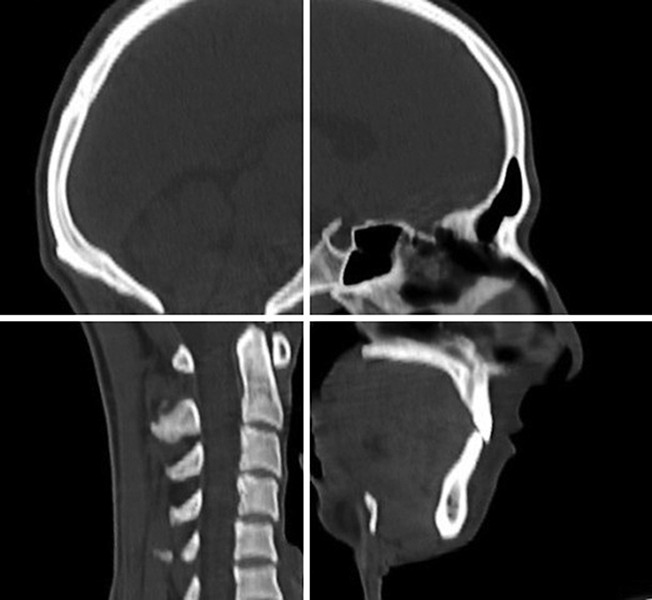
Definition of the spatial coordinate system for the three-dimensional cone beam computed tomography images. The Frankfort horizontal plane and the sagittal plane

Informed consents from the patients and parents were obtained before the study. All cone beam computed tomography (CBCT) scans were taken by a certified radiologist using a Volux 9 3D device under an extended field of view mode (85 × 85 mm). The overall effective radiation dose was 125 mSv, with a 0.35 mm voxel size, a total scanning time of 20 seconds and an effective radiation time of 4.5 seconds. The patients sat upright with the chin supported on an adjustable platform and the Frankfort horizontal plane parallel to the floor while the rotating source detector captured a volumetric image of the patient’s head (Fig. 1). Immediately before scanning, all patients were instructed to keep their teeth in contact throughout the scanning process. Because the nasal cavity contains multiple connecting air cavities, turbinates and rarefactions, a clear segmentation was not possible, and it was excluded from our measurements.

**Fig. 2:**
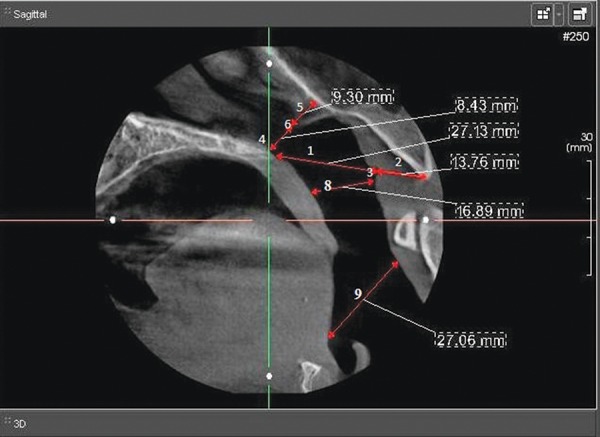
The variables selected for cephalometric evaluation

The following cephalometric measurements were selected^10^ (Fig. 2):

*PNS-AD1:* Lower aerial width, the distance between peripheral nervous system (PNS) and the nearest adenoid tissue measured through the PNS-Ba line (AD1).

*AD1-Ba:* Lower adenoid width, defined as the soft tissue thickness at the posterior nasopharynx wall through the PNS-Ba line.

*PNS-Ba:* Lower airway width, the distance between PNS and Ba - the sum of variables 1 and 2.

*PNS-AD2:* Upper aerial width, the distance between PNS and the nearest adenoid tissue measured through a perpendicular line to S-Ba from PNS (AD2).

*AD2-H:* Upper adenoid width, defined as the soft tissue thickness at the posterior nasopharynx wall through the PNS-H line.

*Hormion (H):* The cephalometric point located near the adenoidal tissue at the cranial base, localized where a perpendicular to S-Ba line crosses the sphenoid bone. The variations of this point are minimal because it is located far from the growing sites.

*PNS-H:* Upper airway width, the distance between PNS and H - the sum of variables 1 and 2.

*McNamara’s upper pharynx dimension:* The minimum distance between the upper soft palate and the nearest point on the posterior pharynx wall.^10^

*McNamara’s lower pharynx dimension:* The minimum distance between the point where the posterior tongue contour crosses the mandible and the nearest point on the posterior.

To assess the measurement error, duplicate measurements of 10 films were made by the same investigator, and the random method described by Dahlberg^21^ was used.

## STATISTICAL ANALYSIS

Error analysis was performed to prevent systematic or random errors. Independent means t-tests and Pearson correlation analysis were used to evaluate sex differences and the correlations among the variables. Statistical significance was established by using a p value less than 0.05.

## RESULTS

**Graph 1:**
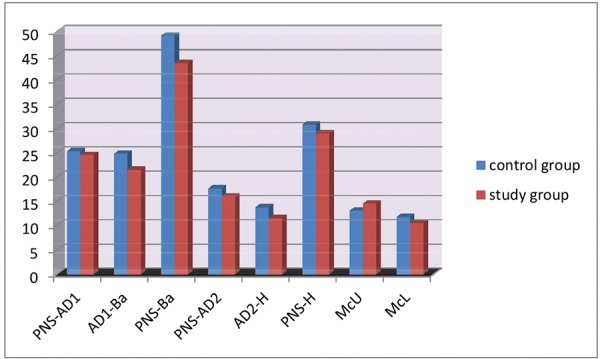
Comparison between control and study group males

**Graph 2:**
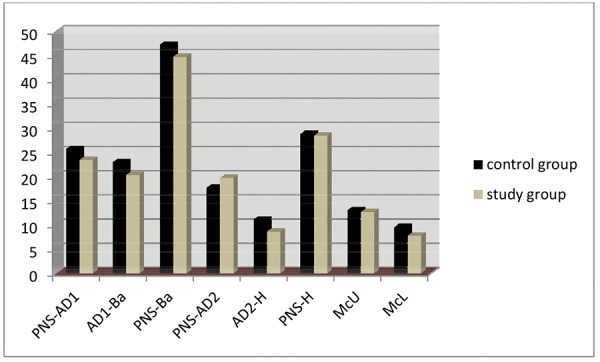
Comparison between control and study group females

**Graph 3:**
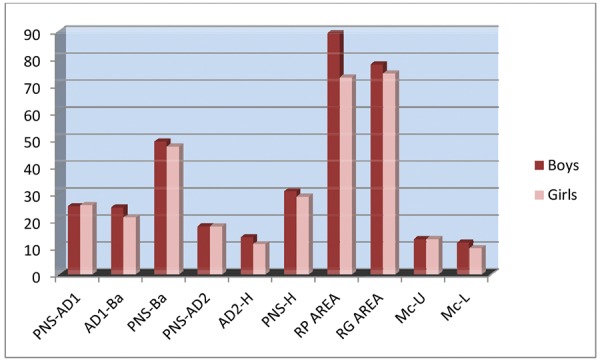
Comparison between males and females

- High standard deviations were noted in the parameter for lower airway width and McNamara lower pharynx width. Since the error method showed reliability near 99%, this means great interindividual variability (Tables 1 to 3).
- Table 1 shows the comparison between nasopharyn-geal characteristics of boys in the control and study groups. Here the lower adenoid width (AD1-Ba), lower airway width (PNS-Ba) and upper airway width (PNS-H) are significantly greater in boys with class I malocclusion (Graph 1).
- Table 2 shows the comparison between nasopharyn-geal characteristics of girls in the control and study groups. Here the lower adenoid width (AD1-Ba), lower airway width (PNS-Ba), upper aerial width (PNS-AD2), upper adenoid width (AD2-H) and McNamara lower airway (Mc-L) are significantly greater in girls with class I occlusion (Graph 2).
- Table 3 shows the comparison between nasopharyn-geal characteristics of boys and girls with class I malocclusion. Here lower adenoid width (AD1-Ba), upper adenoid width (AD2-H) and McNamara lower airway (Mc-L) are significantly greater in boys than in girls with class I malocclusion (Graph 3).

**Table 1:** **Table 1:** Comparison between control and study group males

		*Control group*							*Study group*									
		*Mean*		*SD*		*SE*			*Mean*		*SD*		*SE*		*t-value*		*p-value*	
PNS-AD1		25.33		2.37		0.43			24.5		1.06		0.29		1.793		0.078	
AD1-Ba		24.87		1.85		0.34			21.5		1.36		0.17		7.10		0.0001	
PNS-Ba		49.13		5.32		0.97			43.5		3.12		0.39		1.91		0.015	
PNS-AD2		17.70		3.08		0.56			16		2.01		0.37		3.132		0.063	
AD2-H		13.70		3.41		0.62			11.5		1.57		0.22		6.490		0.071	
PNS-H		30.83		4.05		0.74			29		1.92		0.53		2.047		0.049	
Mc-U		13.03		3.72		0.68			14.5		1.15		0.27		0.344		0.732	
Mc-L		11.77		4.44		0.81			10.5		2.32		0.21		3.987		0.062	

In this table lower adenoid width (AD1-Ba), lower airway width (PNS-Ba) and upper airway width (PNS-H) are significantly greater in boys with class I occlusion. Linear measurements (mm). p-value <0.05 is significant; SD: Standard deviation; SE: Standard error

**Table 2:** **Table 2:** Comparison between control and study group females

		*Control group*							*Study group*									
		*Mean*		*SD*		*SE*			*Mean*		*SD*		*SE*		*t-value*		*p-value*	
PNS-AD1		25.70		2.48		0.45			23.43		1.4		0.10		3.370		0.664	
AD1-Ba		23.00		2.05		0.37			20.27		0.7		0.32		0.437		0.001	
PNS-Ba		47.30		2.94		0.54			44.70		2.12		0.63		2.680		0.010	
PNS-AD2		17.67		4.20		0.77			19.67		2.12		0.74		2.418		0.019	
AD2-H		11.13		1.66		0.30			8.63		0.7		0.56		5.858		0.0001	
PNS-H		28.80		4.34		0.79			28.43		2.8		0.12		0.370		0.712	
Mc-U		13.07		3.18		0.58			12.67		3.5		0.61		0.585		0.561	
Mc-L		9.57		2.47		0.45			7.77		1.7		0.25		3.574		0.001	

This table shows that the lower adenoid width (AD1-Ba), lower airway width (PNS-Ba), upper aerial width (PNS-AD2), upper adenoid width (AD2-H) and McNamara lower airway (Mc-L) are significantly greater in girls with class I occlusion. Linear measurements (mm). p-value <0.05 is significant; SD: Standard deviation; SE: Standard error

**Table 3:** **Table 3:** Comparison between male and female subjects

		*Males*							*Females*									
		*Mean*		*SD*		*SE*			*Mean*		*SD*		*SE*		*t-value*		*p-value*	
PNS-AD1		25.33		2.37		0.43			25.70		2.48		0.45		0.586		0.560	
AD1-Ba		24.87		1.85		0.34			21.00		2.05		0.37		7.664		0.0001	
PNS-Ba		49.13		5.32		0.97			47.30		2.94		0.54		1.652		0.104	
PNS-AD2		17.70		3.08		0.56			17.67		4.20		0.77		0.035		0.972	
AD2-H		13.70		3.41		0.62			11.13		1.66		0.30		3.713		0.0001	
PNS-H		30.83		4.05		0.74			28.80		4.34		0.79		1.875		0.066	
Mc-U		13.03		3.72		0.68			13.07		3.18		0.58		0.037		0.970	
Mc-L		11.77		4.44		0.81			9.57		2.47		0.45		2.371		0.021	

Here lower adenoid width (AD1-Ba), upper adenoid width (AD2-H) and McNamara lower airway (Mc-L) are significantly greater in boys than in girls with class I malocclusion. Linear measurements (mm). p-value <0.05 is significant; SD: Standard deviation; SE: Standard error

The results also suggest that males have larger adenoid width areas than females (Table 3). Males also had greater sagittal thickness of the lower airway.

## DISCUSSION

Nasopharyngeal insufficiency is a major functional problem in patients with CLP who have a narrower pharyngeal airway than do control subjects, as demonstrated in this study. In this context, simple expansion of the maxilla and the mandible might not be the best treatment option for sleep-disordered breathing in patients with CLP, because there is a risk that the nasopharyngeal insufficiency can be exacerbated.

Our data suggest that subjects can vary much more in the lower adenoid and lower aerial region than at the other variables. However, part of this finding might also depend on the position of the centroid, which was defined predominantly in the central region of the airway and used for registration across subjects. Although we did not evaluate the wide variety of problems and issues that can affect the airway, this study does demonstrate that the airway has some variability in shape.

Aboudara et al^22^ found much larger interindividual variations of the volume and area of the upper airway in cephalograms than with CT. Cone beam computed tomography can better assess the cross-sectional dimensions of the airway space than conventional 2D radiography. The drawing of airway circumferences and calculation of cross-sectional areas by computer also greatly reduces operator-dependent bias.

Several authors have reported that the pharyngeal airway space narrows in patients with CLP at the soft palate and the base of the tongue in accordance with mandibular retrognathism.^23-25^ Valiathan et al^26^ suggested that changes in oropharyngeal volume might be attributable to mandibular growth.

Hermann et al^27^ reported that the changes in facial morphology associated with cleft palate result in a small midface and a retruded mandible, leading to a reduced pharyngeal airway space. Liao and Mars^28^ suggested that palatal surgery inhibited the forward displacement of the maxilla and the anteroposterior development of the maxillary dentoalveolus in patients with CLP, but had no detrimental effect on downward displacement of the maxilla or palatal remodeling.

The main purpose of this study was to establish the characteristics of the airway in children with CLP with CBCT. In the present study, we have compared the naso-pharyngeal characters of subjects with CLP with subjects having excellent occlusion. The findings suggested that in subjects with CLP, the lower airway width, upper adenoid width, nasal fossa length, McNamara lower pharynx and total pharyngeal area are decreased and upper aerial width is increased. Also, there was sexual dimorphism in both groups as compared with their male counterparts.

In this study, we opened a new line of investigation that could point to a relationship between skeletal and dental anomalies with airway obstruction and some possible specific respiratory characteristics.

## CONCLUSION

In this study, we described the nasopharyngeal patterns in Indian children with ideal occlusions and CLP. Lower airway width and McNamara lower pharynx width have great interindividual variability in both sexes. There is also sexual dimorphism. Boys have larger adenoid width and adenoid tissue than girls. In the study group, lower adenoid width, lower airway width and upper airway width are reduced when compared to controls.

The narrow pharyngeal airway in patients with CLP might result in functional impairment of breathing in patients. Further investigations are necessary to clarify the relationship between pharyngeal structure and airway function in patients with CLP.

## References

[1] Kirjavainen M, Kirjavainen T (2007). Upper airway dimensions in class II malocclusion. Effects of headgear treatment. Angle Orthod.

[2] MacLean JE, Hayward P, Fitzgerald DA, Waters K (2009). Cleft lip and/or palate and breathing during sleep. Sleep Med Rev.

[3] Imamura N, Ono T, Hiyama S, Ishiwata Y, Kuroda T (2002). Comparison of the sizes of adenoidal tissues and upper airways of subjects with and without cleft lip and palate. Am J Orthod Dentofacial Orthop.

[4] Warren DW, Hairfield WM, Dalston ET, Sidman JD, Pillsbury HC (1988). Effects of cleft lip and palate on the nasal airway in children. Arch Otolaryngol Head Neck Surg.

[5] Hairfield WM, Warren DW, Seaton DL (1988). Prevalence of mouth-breathing in cleft lip and palate. Cleft Palate J.

[6] Drake AF, Davis JU, Warren DW (1993). Nasal airway size in cleft and noncleft children. Laryngoscope.

[7] Rose E, Staats R, Thissen U, Otten JE, Schmelzeisen R, Jonas I (2002). Sleep-related obstructive disordered breathing in cleft palate patients after palatoplasty. Plast Reconstr Surg.

[8] Marcus CL (1997). Clinical and pathophysiological aspects of obstructive sleep apnea in children. Pediatr Pulmonol Suppl.

[9] Oosterkamp BCM, Remmelink HJ, Pruim GJ, Hoekema A, Dijkstra PU (2007). Craniofacial, craniocervical, and pharyngeal morphology in bilateral cleft lip and palate and obstructive sleep apnea patients. Cleft Palate Craniofac J.

[10] Martin O, Muelas L, Vinas MJ (2006). Nasopharyngeal cephalometric study of ideal occlusions. Am J Orthod Dentofacial Orthop.

[11] Baumrind S, Frantz RC (1971). The reliability of head film measurements. 2. Conventional angular and linear measures. Am J Orthod.

[12] Huang J, Bumann A, Mah J (2005). Three-dimensional radiographic analysis in orthodontics. J Clin Orthod.

[13] Mah J, Hatcher D (2004). Three-dimensional craniofacial imaging. Am J Orthod Dentofacial Orthop.

[14] Maki K, Inou N, Takanishi A, Miller AJ (2003). Modeling of structure, quality, and function in the orthodontic patient. Orthod Craniofac Res.

[15] Hatcher DC, Aboudara CL (2004). Diagnosis goes digital. Am J Orthod Dentofacial Orthop.

[16] Araki K, Maki K, Seki K, Sakamaki K, Harata Y, Sakaino R, Okano T, Seo K (2004). Characteristics of a newly developed dento-maxillofacial x-ray cone beam CT scanner (CB MercuRay): system configuration and physical properties. Dentomaxillofac Radiol.

[17] Cevidanes LH, Styner MA, Proffit WR (2006). Image analysis and superimposition of 3-dimensional cone-beam computed tomography models. Am J Orthod Dentofacial Orthop.

[18] Cevidanes LH, Bailey LJ, Tucker SF, Styner MA, Mol A, Phillips CL, Proffit WR, Turvey T (2007). Three-dimensional cone-beam computed tomography for assessment of mandibular changes after orthognathic surgery. Am J Orthod Dentofacial Orthop.

[19] Ogawa T, Enciso R, Memon A, Mah JK, Clark GT (2005). Evaluation of 3D airway imaging of obstructive sleep apnea with cone-beam computed tomography. Stud Health Technol Inform.

[20] Ogawa T, Enciso R, Shintaku WH, Clark GT (2007). Evaluation of cross-section airway configuration of obstructive sleep apnea. Oral Surg Oral Med Oral Pathol Oral Radiol Endod.

[21] Dahlberg G (1940). Statistical methods for medical and biological students..

[22] Aboudara CA, Hatcher D, Nielsen IL, Miller A (2003). A three-dimensional evaluation of the upper airway in adolescents. Orthod Craniofac Res.

[23] Jena AK, Singh SP, Utreja AK (2010). Sagittal mandibular development effects on the dimensions of the awake pharyngeal airway passage. Angle Orthod.

[24] Muto T, Yamazaki A, Takeda S (2008). A cephalometric evaluation of the pharyngeal airway space in patients with mandibular retrognathia and prognathia, and normal subjects. Int J Oral Maxillofac Surg.

[25] Tsai HH (2007). Developmental changes of pharyngeal airway structures from young to adult persons. J Clin Pediatr Dent.

[26] Valiathan M, El H, Hans MG, Palomo MJ (2010). Effects of extraction versus non-extraction treatment on oropharyngeal airway volume. Angle Orthod.

[27] Hermann NV, Kreiborg S, Darvann TA, Jensen BL, Dahl E, Bolund S (2002). Early craniofacial morphology and growth in children with unoperated isolated cleft palate. Cleft Palate Craniofac J.

[28] Liao YF, Mars M (2005). Long-term effects of lip repair on dentofacial morphology in patients with unilateral cleft lip and palate. Cleft Palate Craniofac J.

